# Pain Perception and Anxiety Levels during Menstrual Cycle Associated with Periodontal Therapy

**DOI:** 10.1155/2014/472926

**Published:** 2014-10-12

**Authors:** Nikhat Fatima, P. Raja Babu, Vidya Sagar Sisinty, Bassel Tarakji

**Affiliations:** ^1^Department of Periodontics, Al-Farabi College, Riyadh, Saudi Arabia; ^2^Department of Periodontics, Kamineni Institute of Dental Sciences, Narketpally, India; ^3^Department of Oral and Maxillofacial Sciences, Faculty of Dentistry, Al-Farabi College, Riyadh, Saudi Arabia

## Abstract

*Objectives*. To compare the pain perception and anxiety levels of female patients undergoing scaling and root planing during menstrual (perimenstrual) period with those observed during postmenstrual period. *Materials and Methods*. This was a single blind study, with a split-mouth design. Forty-four women with chronic periodontitis with regular menstrual cycles were subjected to complete Corah's Dental Anxiety Scale (DAS) during their first debridement visit. Patients were randomly selected to undergo their first debridement visit during either their menstrual or postmenstrual period. Scaling was performed under local anesthesia in bilateral quadrants of patients during the periods. Visual Analogue Scale (VAS) was used to score pain levels for each quadrant after performing scaling and root planing. *Results*. Increase in pain perception among females during their menstrual or perimenstrual period was significantly greater than their postmenstrual period (*P* < 0.05). It is observed that women whose first appointment was given in perimenstrual period had more pain (VAS) (*P* = 0.0000) when compared to those women whose first appointment was given in postmenstrual period. *Conclusion*. Females in their menstrual period demonstrated higher pain responses and high anxiety levels to supra- and subgingival debridement. This increase in the pain levels of women during their menstrual period was statistically significant. If the appointments are given in postmenstrual period, women feel less pain.

## 1. Introduction

It is estimated that some 5% to 20% of any population suffers from severe generalized periodontitis [1]. Periodontitis is defined as an inflammatory disease of the supporting tissues of the teeth caused by specific microorganisms resulting in progressive destruction of the periodontal ligament and bone loss [2]. If untreated, this chronic inflammatory disease can lead to tooth loss affecting the oral health related quality of life (QOL) [3].

When we consider the gender basis of periodontal diseases, there is evidence to support the higher prevalence of destructive periodontal disease in men than women. The important factor to be considered is that women still have varied periodontal conditions due to hormonal fluctuations in their various phases of life [4].

During the treatment phases of periodontitis (this includes mechanically removing bacterial biofilm), scaling and root planing patients feel that the procedure is more painful [5, 6]. There is increased evidence in literature that indicates males and females perceive pain differently when they were subjected to experimental or clinical pain. Several factors have been suggested to explain the differences with the most common difference being the sex hormones. The fluctuating hormones affect women's perception of pain as well as increase in anxiety [7, 8].

From oral health point of view, there have been studies which have been conducted to demonstrate degree of pain and anxiety experienced by patients during probing and scaling and their perception of pain was directly proportional to anxiety levels [9].

Özçaka et al. [9] in their pilot study with the small sample size of 20 Turkish women demonstrated the effect of menstrual cycle on pain experience during periodontal therapy. Since then no study has been done, especially in a developing country, so we decided to conduct a study in Indian women in 44 patients to determine the effect of the menstrual cycle and role of anxiety which plays any effect on pain perception.

## 2. Materials and Methods

A total of forty-four (44) female subjects between the ages of 20 and 38 (mean 27 years) were recruited from the nursing, medical, and dental college and also the female patients visiting Department of Periodontics, Kamineni Institute of Dental Sciences, Narketpally, Nalgonda District. This study was approved by Ethical Committee of Kamineni Institute of Medical Sciences and Dental Sciences.

### 2.1. Objectives

The objective of this study is to compare the pain perception of female patients and their anxiety levels when undergoing periodontal debridement during

1. menstrual phases,
2. postmenstrual phase.

#### 2.1.1. Patient Selection Criteria

- Inclusion criteria:
  1. patients should have chronic periodontitis, with at least five teeth in each quadrant;
  2. at least 3 teeth in each quadrant were required to have a probing pocket depth of 4 mm or greater;
  3. none of the subjects had received periodontal debridement within the preceding 12 months;
  4. patients should have regular menstrual cycle.
- Exclusion criteria:
  1. patients on oral contraceptives;
  2. patients on antidepressants and analgesics;
  3. patients with systemic disease.

### 2.2. Design of the Study

The total of forty-four patients was selected for the study. This study was a single blind study, with a split-mouth design, where patients were aware of their menstrual status but supporting examiner was not aware of the patients' menstrual status.

Based on clinical findings, patients who exhibited moderate-to-advanced chronic periodontitis were included in the study. Orthopantogram (OPG), probing pocket depth (PPD), clinical attachment loss (CAL), gingival index (GI) [10], periodontal index (PI) [10], and bleeding on probing (BOP) [10] were recorded. Patients' menstrual history is taken and those who reported having regular menstrual cycles (i.e., the length of their cycle varied by no more than 3 days) for the last 12 months were eligible for the study. A menstrual cycle is conventionally defined as the time from the beginning of one menstrual flow (day 1) to the beginning of the next [11]. Although there is a great deal of variability in menstrual cycle length between women and within individual women over time [12] the prototypical menstrual cycle is usually described for heuristic purposes as 28 days in length. Patients who exhibited probing pocket depth 4 mm or greater, and who had at least 5 teeth in each quadrant, and severity of periodontal disease was similar were included in the study. At least three teeth in each quadrant were required to have a probing pocket depth of 4 mm or greater.

The patients who are on antidepressants and analgesics were excluded from the study because antidepressants and analgesics may have an effect on pain threshold [5]. And it was made sure that none of the subjects had received periodontal debridement within the preceding 12 months. Corah's Dental Anxiety Scale (DAS) [13] and Visual Analog Scale (VAS) [14] numerical were used to assess the anxiety and pain perception, respectively. Corah's Dental Anxiety Scale was translated into local language (Telugu) for those subjects who were comfortable in their local language.

### 2.3. Clinical Procedures

The scheduling of the first treatment appointment was done randomly. Patients took their first appointment during their first 3 days of menstrual period or in perimenstrual period between 3 and 4 days before the menstrual period. Some patients took their first appointment one week after the menstruation, that is, in postmenstruation. Supragingival and subgingival scaling was performed under local anesthesia. All patients received infiltrative anesthesia containing lidocaine 2% with adrenaline 1 : 80000 [15].

This study was performed with a split-mouth design, and another debridement took place according to the menstrual phase of the patient.

The treatment was standardized by noting the time taken for debridement, the amount of local anesthesia used, the number and type of teeth to be debrided at each visit were equalized.

Before the start of treatment, in their first debridement visit, patients were asked to complete Corah's Dental Anxiety Scale (DAS) that was translated in Telugu.

This was a single-blinded study. The supporting investigator was not informed about the menstrual status of the subjects. However, the patients were aware of the study. Supragingival and subgingival debridement was carried out by the same investigator.

After applying local anesthesia, supragingival and subgingival scaling was performed. When the effect of local anesthesia wore off after 2 hours, patients started to perceive pain; they were asked to complete Visual Analogue Scale (VAS) numerical. The scale starts from “0” to “100”; the starting point indicates “no pain” and the end point indicates “intolerable pain.” Some patients left the clinic taking VAS form and returned it in their second visit; the rest of the patients completed the procedure within the clinic.

### 2.4. Data Analysis

A total of 44 female patients participated in the study aging between 20 and 38 years. (mean age 27). Perimenstrual and Postmenstrual Dental Anxiety Scale (DAS) and Visual Analogue Scale (VAS) scores of females were collected and analysed by the Wilcoxon signed rank test (see Tables 1 and 2). Comparison of the possible effects of the order of treatment time (perimenstrual or postmenstrual) was evaluated by Wilcoxon's signed rank test.

**Figure 1:**
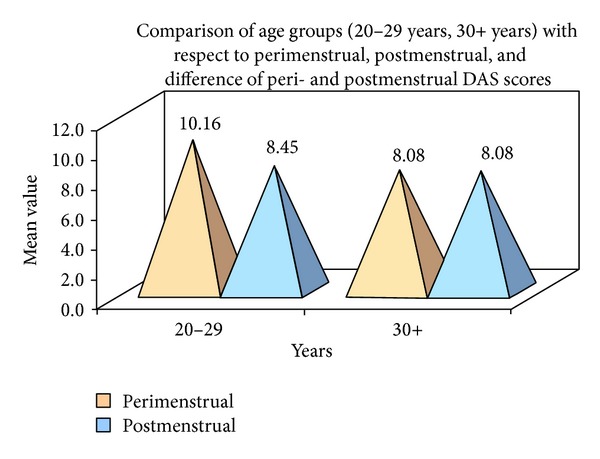
Comparison of age groups (20–29 years, 30+ years) with respect to perimenstrual, postmenstrual, and difference of peri- and postmenstrual DAS scores.

Comparison of perimenstrual and postmenstrual PPD (%) scores was done by student's paired *t*-test. Comparison of age groups (20–29 years, 30+ years) with respect to perimenstrual, postmenstrual, and difference of pre- and postmenstrual DAS and VAS scores was done by Mann-Whitney *U* test (see Figure 1).

Frequency distribution of patients' Dental Anxiety Scale and VAS pain responses to periodontal treatment was analyzed.

## 3. Results

It has been observed that the increase in anxiety among females in their perimenstrual period was significantly greater than their postmenstrual period (Wilcoxon's signed rank test) (DAS) (*P* < 0.0200).

The increase in pain perception among females in their perimenstrual period was significantly greater than their postmenstrual period (Wilcoxon's signed rank test) (VAS) (*P* < 0.05).

Perimenstrual PPD% was (mean scores) 53.50 and postmenstrual PPD% was (mean score) 52.88 (*P* > 0.0461) significant showing increase in pocket depth during perimenstrual period. And student's paired *t*-test was (2.0537) which is highly significant. It has been observed in the study that perimenstrual bleeding on probing was more (mean score 50.72) than postmenstrual bleeding on probing (mean score 49.57).

In this study women showed appreciable oral symptoms during menstruation. In perimenstruation, the mean GI score was 0.8091 compared to postmenstruation GI score (mean score 0.7977).

Statistically significant associations were observed between age and anxiety. Women who are aged 30 and above have demonstrated more anxiety in their perimenstruation phase compared to women who are below 30 (*P* < 0.01). No statistically significant associations were observed between age and pain perception.

Comparison of order of treatment time, perimenstrual to postmenstrual, with respect to DAS is analyzed by using Mann-Whitney *U* test. It is observed that women whose first appointment was given in perimenstrual period had more anxiety (DAS) (*P* < 0.0041) compared to those women whose first appointment was given in postmenstrual period.

Comparison of order of treatment time, perimenstrual to postmenstrual, with respect to VAS is analyzed by using Mann-Whitney *U* test. It is observed that women whose first appointment was given in perimenstrual period had more pain (VAS) (*P* < 0.0000) compared to those women whose first appointment was given in postmenstrual period.

## 4. Discussion

A woman's life is continuously affected by reproductive hormones, in puberty, pregnancy, and menopause. Woman's oral health needs can also change at these times, thus affecting their dental treatment plans. In the past, research on women's health has been unfairly neglected and only recently research and health agencies decided to change this [16]. Therefore, recent research has identified many interesting and important differences between genders in terms of oral health and pain perception.

Female reproductive hormones play important role in pain perception and response. A menstrual cycle is conventionally defined as the time from the beginning of one menstrual flow (day 1) to the beginning of the next [11]. Although there is a great deal of variability in menstrual cycle length between women and within individual women over time [12], the prototypical menstrual cycle is usually described for heuristic purposes as 28 days in length. Gynecologists divide the menstrual cycle into phases based on physiological events. The monthly reproductive cycle has two phases. The first phase is referred to as the follicular phase. Levels of follicle-stimulating hormone (FSH) are elevated and estradiol (*E*_2_) the major form of estrogen is synthesized by the developing follicle and peaks approximately 2 days before ovulation.

In the second phase which is called luteal phase, the developing corpus luteum synthesizes both estradiol and progesterone. The corpus luteum involutes, ovarian hormones level drops, and menstruation ensue. It has been postulated that ovarian hormones may increase inflammation in gingival tissues and exaggerate the response to local irritants and increase in oral symptoms during menses [17]. Miyagi et al. found that the chemotaxis of polymorphonuclear leucocytes was enhanced by progesterone but reduced by estradiol. Testosterone did not have measurable effect on polymorphonuclear leucocytes chemotaxis [18].

It is now generally accepted that males and females exhibit important differences in their pain experiences [18–20]. For example, epidemiological studies indicate that females report more pain experiences and more negative responses to pain compared to males [7]. Furthermore, clinically based research suggests that there are important gender differences in susceptibility to pain-related diseases, analgesic effectiveness, and recovery from anaesthesia [21, 22].

Finally, experimental pain induction studies reveal that females consistently exhibit lower thresholds and tolerance to a wide range of noxious stimuli [23, 24]. A number of psychological factors, including anxiety, may be sources of variance in how men and women perceive pain. Women are often found to experience more transitory and dispositional anxiety than men [25–28].

Anxiety is believed to disrupt the interpretation of stimuli, resulting in altered perception. It is thought that anxiety disrupts the experience of pain by influencing the cognitive processing of nociceptive information [29]. Laboratory induced general anxiety [30] and pain-specific anxiety [31] have both been found to correspond with increased sensitivity to painful stimulation.

The patients who are on antidepressants and analgesics were excluded from the study because antidepressants and analgesics may have an effect on pain threshold [5]. It is concluded that tricyclic antidepressants have differential hypoalgesic effect on different human experimental pain tests [32].

The purpose of this study was to determine differences between females' pain levels during their perimenstrual versus their postmenstrual phase by performing debridement under local anesthesia.

During this study it has been observed that in campus nursing, dental, and medical students were more cooperative and followed scheduled appointments regularly. The general female patients who visited our department did not keep the track of their menstrual cycle and did not fulfill the study requirement. Six female patients did not complete their second visit.

Corah's Dental Anxiety Scale (DAS) was translated into Telugu for those who are comfortable with expressing their feelings in their local language. We preferred the VAS which requires minimal linguistic skills and is therefore easier for patients to use in describing their pain. VAS can be quantified and allows for comparison of patients on a scale of 0–100.

In this study the increase in pain perception among females in their perimenstrual period was significantly greater than their postmenstrual period (Wilcoxon's signed rank test) (VAS) (*P* = 0.0000) and also anxiety was greater in perimenstrual period (DAS) (*P* < 0.0200). It is also observed that women whose first appointment was given in perimenstrual period had more anxiety (DAS) (*P* < 0.0041) and more pain (VAS) (*P* = 0.0000) compared to those women whose first appointment was given in postmenstrual period. With respect to this result, we found that patients in their perimenstrual period demonstrated higher pain responses to supra- and subgingival debridement than they did in their postmenstrual period. This increase in the pain levels of women during their menstrual period was statistically significant. There was no statistically significant correlation between the DAS and the VAS. Spearman's “*P*” was 0.2429. Statistically significant associations were observed between age and anxiety. Women who are aged 30 and above have demonstrated more anxiety in their perimenstruation phase compared to women who are below 30. No statistically significant associations were observed between age and pain perception.

This study also confirms results of the other study by Ozgun Ozcaka and collegues.

Gender based medicine and gender based treatment are going to become the norm of the day. By knowing the menstrual cycles of their female patients, periodontists may wish to schedule mechanical periodontal therapy; this will instill positive attitude in female patients regarding dentistry in general and periodontal therapy in particular and improves the comfort level and cooperation from female patients.

## 5. Conclusion

This study addressed the specific problem of female patients undergoing periodontal therapy in their perimenstrual and postmenstrual period. It has been observed that women perceive more pain during perimenstrual period than in their postmenstruation period. Periodontists can make women more comfortable and cooperative by scheduling their appointment according to their cycle.

## Figures and Tables

**Table 1:** Comparison of perimenstrual and postmenstrual DAS scores by Wilcoxon matched pairs test by ranks.

Menstrual	Mean	Std.Dv.	Mean Diff.	SD Diff.	% of change	*Z* value	*P* value
Perimenstrual	9.5455	3.0534					
Postmenstrual	8.3409	3.5955	1.2045	3.4071	12.6190	2.3258	0.0200∗

*Significant at 5% level of significance (*P* < 0.05).

**Table 2:** Comparison of perimenstrual and postmenstrual VAS scores by Wilcoxon matched pairs test by ranks.

Menstrual	Mean	Std.Dv.	Mean Diff.	SD Diff.	% of change	*Z* value	*P* value
Perimenstrual	57.9318	11.7263					
Postmenstrual	40.1136	10.7297	17.8182	7.8066	30.7572	5.7592	0.0000∗

*Significant at 5% level of significance (*P* < 0.05).
